# Vulvovaginal candidiasis: knowledge, practices and occurrence among pregnant women receiving antenatal care in a teaching hospital, Ghana

**DOI:** 10.3389/fgwh.2025.1647140

**Published:** 2025-08-25

**Authors:** Florence Shine Edziah, Princess Ruhama Acheampong, Philip Apraku Tawiah, Cedric Dzidzor Amengor, Godsway Edem Kpene, Grace Otobea Amponsah, Priscilla Appiah Baffoe, Georgina Korankye, John Gameli Deku

**Affiliations:** ^1^ Department of Health Promotion & Disability Studies, School of Public Health, College of Health Sciences, Kwame Nkrumah University of Science and Technology, Kumasi, Ghana; ^2^ Department of Medical Laboratory Sciences, School of Allied Health Sciences, University of Health and Allied Sciences, Ho, Ghana; ^3^ Department of Occupational and Environmental Health & Safety, School of Public Health, College of Health Sciences, Kwame Nkrumah University of Science and Technology, Kumasi, Ghana; ^4^ Department of Pharmaceutical Chemistry, School of Pharmacy, University of Health and Allied Sciences, Ho, Ghana; ^5^ Department of Obstetrics and Gynecology, Mothers Clinic, Ho Teaching Hospital, Ho, Ghana; ^6^ Department of Medical Microbiology, School of Medicine, College of Health Sciences, University of Ghana, Legon, Ghana

**Keywords:** candidiasis, pregnant women, antibiotic use, knowledge, practices

## Abstract

**Background:**

Vulvovaginal Candidiasis (VVC) is a condition commonly caused by *Candida albicans*. It is the second most common infection of the female genitalia affecting many women worldwide. Studies have identified unhealthy genital care practices to be associated with the infection among women including expectant mothers. Knowledge of the various signs and symptoms is crucial for early detection, reporting, and treatment. Good knowledge may influence healthy practices, limiting the infection and its complications. This study assessed the knowledge, practices and occurrence of Vulvovaginal candidiasis among pregnant women accessing antenatal care at a teaching hospital in Ghana.

**Methods:**

A cross-sectional study was conducted among 336 pregnant women receiving antenatal care at the Ho Teaching Hospital. A structured questionnaire was used to assess their knowledge of the infection and practices on vaginal hygiene. The hospital records of these participants were further checked to verify the occurrence of the infection. Data were analyzed using Stata version 16. Analysis to identify associations between outcome variables and risk factors as well as significance level was carried out.

**Results:**

Out of the 336 gestational mothers involved in the study, 27.1% were found to have been diagnosed with candidiasis at the time of the study. Pregnant women who usually use antibiotics had 2.25 increased odds of developing VVC compared to those who do not [OR:2.25 95CI:1.33–3.79; *p*-value = 0.003]. Although, good knowledge was recorded among a greater percentage of the study participants on the various signs and symptoms including vaginal discharge and its abnormalities, there was however, a poor knowledge on the causative organism, as 77.4% indicated that the infection was caused by other agents rather than fungi.

**Conclusion:**

The occurrence of VVC was elevated in the study jurisdiction. Frequent antibiotic use was found to be significantly associated with the occurrence of the infection.

## 1 Introduction

Vulvovaginal candidiasis (VVC) has been reported globally as a condition affecting a significant number of women. It is a global burden among women with 75% experiencing an episode in their lifetime (1). An investigation carried out on the prevalence of recurrent vulvovaginal candidiasis in five European countries and the U.S revealed that, over 40% of women reported at least four acute episodes of VVC in a 12-month period (2). Also, studies show a rise in VVC in Asia and Africa (3). In South Asia, a prevalence of 55.5% was recorded among pregnant women receiving care at a gynecological and obstetrics unit in a tertiary healthcare facility (4) whereas, in Africa, a recent study found a total prevalence of 43.3% among pregnant women in South East Nigeria (5).

In Ghana, 36.5% prevalence was recorded among pregnant women in the middle belt (6) and 30.7% in the southern belt (7). Gestational mothers experience this condition due to the change in pH and sugar content of vaginal secretions resulting in the overgrowth of the causative organism (8). Apart from the biological causal pathway, lifestyle and hygiene practices have also been reported to influence the infection rate greatly (9).

Some pregnant women indulge in unhealthy practices as a result of inadequate knowledge, enhancing their vulnerability to the disease. A study among antenatal care attendees on genital hygiene practices identified some risky activities such as the use of antiseptic solutions for genital cleansing and the use of synthetic underwear. Other findings have identified practices such as not changing underwear daily, wearing inadequately-dried underwear and using herbal combinations to wash the private part for easy baby delivery (10). Again, a survey on the analysis of genital hygiene behavior among women revealed that most pregnant women undergo vaginal douching (11).

Improper antibiotic use, which is an associated factor of increased prevalence has also been identified among gestational mothers (12). Among the risk factors, antibiotic use was ranked highest followed by sexual intercourse, humid weather and use of feminine hygiene products (13). Antibiotics destroy the protective bacteria flora in the vagina and allow the overgrowth of yeast. A previous study indicated that 28%–33% of women who undergo antibiotic therapy experience symptomatic genital candidiasis (14). Short courses of oral antibiotic therapy have been identified with increased prevalence of symptomatic VVC (15, 16).

A good knowledge may influence healthy practices and enhance preventive measures hence limiting the rate of infection. Also, knowledge of the various signs and symptoms is crucial for early detection, reporting, and treatment. Knowledge on various vaginal discharges is key for the detection of the infection. A common sign associated with the infection is abnormal discharge. however, a study by Varghese (17) found that, abnormal vaginal discharge was perceived by most women as normal. This indicates a challenge in the detection of normal vaginal discharge. A common gynecological complain among pregnant women is the inability to differentiate between normal and abnormal vaginal discharge leaving a gap in knowledge (18).

Most studies in Ghana (6, 7) focused on prevalence of the disease. However, there was no further investigation to identify the predisposing factors associated with such rate of occurrence. It is, therefore, relevant to assess the knowledge and practices of these women for appropriate intervention measures, hence this study seeks to assess the relationship between knowledge, practices and occurrence of VVC among pregnant women at the Ho Teaching Hospital of Ghana.

## 2 Materials and methods

### 2.1 Study design

A cross-sectional study design was employed for this study. The study involved administration of questionnaire to assess participants’ knowledge and practices that could expose them to the infection. Data was collected from 5th September 2023 to 12th October 2023.

### 2.2 Study participants and setting

The study participants included pregnant women who visited the antenatal care clinic (ANC) of the Ho Teaching Hospital for health care services. The study site is located in the Volta Region, Ho, Ghana and serves as the major referral center in the region with 340-bed capacity. The hospital offers specialized healthcare services, including cardiothoracic care, intensive care, dialysis, dental services, operation smile, and operation hernia. It also provides daily obstetrics and gynecology services, pharmaceutical care, laboratory services, and eye care. The ANC unit, staffed by 27 midwives, delivers outpatient services and in-patient care on daily basis.

Pregnant women in the municipality and beyond visit the facility to receive care. On the average, 20 pregnant women report to the unit on a daily basis.

### 2.3 Sample size determination

The Cochran sample size calculation formula was used to obtain the appropriate sample size. A minimum sample size of 329 was calculated based on a 95% confidence level with the prevalence of a recent study in the Ho Municipality (30.7%) (7). The minimum sample size (*n*) = z^2^pd/d^2^, where z = Standard deviation (1.96) at 95% confidence limit, *P* = Prevalent rate = 31%, q = 1-*P* = 1%–0.31% = 0.69, d = Error margin = 5%) However, a total sample size of 336 was used for the study.

### 2.4 Sampling procedure

A simple random sampling method was used to recruit pregnant women receiving antenatal care at the facility. All participants were gestational mothers who agreed to partake in the study. Each received a pre-enrollment briefing about the study, and an informed written consent was obtained. Participants were provided with questionnaires to obtain information on socio-demographic characteristics, knowledge and practices relating to the infection.

### 2.5 Inclusion and exclusion criteria

#### 2.5.1 Inclusion criterion

Pregnant women who provided informed consent after obtaining information on the study's objectives, risks, and benefits.

#### 2.5.2 Exclusion criteria

Pregnant women who declined participation and those with confirmed mental health problem were excluded.

### 2.6 Data collection

A pre-enrollment briefing was carried out and willing participants provided a written consent to partake in the study. Prior to the collection of the data, an exploratory review was done by experts in the microbiology and public health domain to ensure content validity.

Again, a pre-testing of the questionnaire was done on pregnant women in the Ho metropolis to ensure that the questionnaire was clear and easy to understand. The closed ended questionnaire consisted of three sections, and obtained data on sociodemographic characteristics (age, sex, marital status, level of education and occupation), knowledge on VVC and practices relating to the infection. The knowledge component was made up of eleven questions addressing fundamental aspects of the infection, signs and symptoms, preventive strategies and treatment option for candidiasis. The practice component consisted of seven questions that evaluated hygiene practices and the use of antibiotics.

Participants completed the questionnaires in a private setting to ensure confidentiality. Each questionnaire was completed within 10 minutes. Again, the hospital records of the participants were further checked to verify the occurrence of candidiasis. The questionnaire was administered in English. However, it was translated to the local language (Ewe) for those participants who could not read and understand the English. The frequency of each procedure was assessed by calculating the number and percentage of the respondents for each response category. The study was approved by the relevant ethical review board before the commencement of data collection.

### 2.7 Data management and analysis

The administered questionnaires were appropriately coded and entered into Microsoft Excel 2013 for cleaning. Upon validation, the cleaned data was exported to STATA (statistical analysis software) Version 16 for statistical analysis.

The various responses on knowledge and practices were recorded. Prevalence of vulvovaginal candidiasis was determined based on evidence from their medical records on laboratory investigations establishing the presence of the infection.

Categorical variables were presented as frequencies and percentages, while continuous variables were represented as means, median, standard deviations or interquartile ranges. “Bivariate analysis was performed to examine the correlation between some demographic characteristics, practices and the outcome of interest at a significance level of *p* < 0.05.

### 2.8 Ethical consideration

To adhere to ethical guidelines, ethical approval (Ref: CHRPE/AP/703/23) was obtained from the Committee on Human Research, Publication and Ethics of Kwame Nkrumah University of Science and Technology before commencing the research. Additionally, written permission was sought from the management of Ho Teaching Hospital to conduct the study.

## 3 Results

### 3.1 Socio-demographic characteristics of gestational mothers

Table 1 below summarizes the socio-demographic characteristics of gestational mothers receiving antenatal care at the Ho Teaching Hospital at the time of the study. Out of the 336 participants, 196 (58.33%) aged 30–39 years. In relation to education and employment status, 156 (46.4%) each out of the total respondents had tertiary education and were self-employed. The greatest number of these expectant mothers, 197 (58.63%) were in the third trimester of their pregnancy. Majority of them were Christians 317 (94.4%) and married 263 (78.3%).

**Table 1:** Socio-demographic characteristics of gestational mothers.

Characteristics	Frequency	Percentage
Age category (years)		
16–29	126	37.5
30–39	196	58.3
40 and above	14	4.2
Marital status		
Single	51	15.2
Married	263	78.3
Co-habiting	22	6.5
Religion		
Christianity	317	94.3
Islamic	19	5.7
Level of education		
No formal education	6	1.8
Primary	81	24.1
Secondary	93	27.7
Tertiary	156	46.4
Employment status		
Unemployed	26	7.7
Student	17	5.1
Government worker	112	33.3
Private firm	25	7.4
Self employed	156	46.4
Gestational period		
First trimester	43	12.8
Second trimester	96	28.6
Third trimester	197	58.6

### 3.2 Knowledge of pregnant women on vulvovaginal candidiasis

Table 2 below summarize the level of knowledge among the study participants on the infection. Generally, a good knowledge was recorded among the greater percentage on major signs and symptoms, however, it was described as a bacterial infection by 209 (62.2%). Vomiting and fatigue was rightly indicated by 298 (88.7%) of the respondents as not being a sign/symptom associated with the infection. Burning sensation and pain in the vagina were rightly indicated as symptoms by 255 (75.89%) and 242 (72.02%) respectively. Vaginal itching was indicated by 308 (91.67%) as a symptom of candidiasis. A cheese-like vaginal discharge with odor was indicated by majority, 300 (89.29%) as the abnormal discharge associated with the infection. Almost all the respondents, 308 (91.67%) viewed Vulvovaginal candidiasis as a possible infection that can occur in pregnancy.

**Table 2:** Knowledge of pregnant women on vulvovaginal candidiasi**s.**

Characteristics	Frequency	Percentage
Type of infection		
Fungal	76	22.6
Bacterial	209	62.2
Viral	21	6.3
Parasitic	30	8.9
Signs/symptoms of VVC		
Vomiting		
No	298	88.7
Yes	38	11.3
Fatigue		
No	283	84.2
Yes	53	15.8
Burning sensation		
No	81	24.1
Yes	255	75.9
Pain in vagina		
No	94	28.0
Yes	242	72.0
Vagina itching		
No	28	8.3
Yes	308	91.7
Normal vaginal discharge		
No	269	80.1
Yes	67	19.9
Abnormal discharge		
No	43	12.8
Yes	293	87.2
Pain/discomfort during sex		
No	106	31.5
Yes	230	68.5
Associated vaginal discharge		
Normal without odor	36	10.7
Cheese-like with odor	300	89.3
View on VVC in pregnancy		
VVC cannot occur in pregnancy		
False	308	91.7
True	28	8.3

### 3.3 Practices of pregnant women towards the occurrence of VVC

In Table 3 below, the summary of some practices among gestational mothers of this study can be found. The use of feminine hygiene washes 228 (85.71%) and daily perineal pads 223 (66.37) was identified among the expectant mothers. Out of the total number of respondents, 302 (89.88%), 321 (95.54%), 244 (72.62%) and 208 (64.20%) changed their underwear daily, preferred cotton as fabric for their underwear, dried panties under the sun after washing and ironed their underwear respectively. Frequent antibiotic usage was indicated by 134 (39.88%) of the study participants.

**Table 3:** Practices of pregnant women towards the occurrence of VVC.

Characteristics	Frequency	Percentage
Use feminine hygiene wash		
No	288	85.7
Yes	48	14.3
Use daily perineal pad		
No	223	66.4
Yes	113	33.6
Change underwear daily		
No	34	10.1
Yes	302	89.9
Preferred fabric type for underwear		
Linen	7	2.1
Cotton	321	95.5
Nylon	8	2.4
Where panty is dried after washing		
Bathroom	35	10.4
Bedroom	57	17.0
Under the sun	244	72.6
Iron underwear before wearing		
No	208	64.2
Yes	116	35.8
Usually takes antibiotics		
No	202	60.1
Yes	134	39.9

### 3.4 Occurrence of VVC among the gestational mothers

A prevalence of 27.1% (91/336) was observed among the expectant mothers receiving antenatal care at the Teaching Hospital.

### 3.5 Association between socio-demographic characteristics and the occurrence of VVC

Table 4 below describes the association between the various socio-demographic characteristics and the prevalence of the infection. There was no significant association between the socio-demographic characteristics and the occurrence of the infection.

**Table 4:** Association between occurrence and socio-demographic variables.

Characteristics	Total (%)	Confirmed cases		X^2^	*p*-value
		Negative	Positive		
Age					
16–29	126 (37.5)	84 (66.67)	42 (33.33)	4.02	0.134
30–39	196 (58.33)	150 (76.53)	46 (23.47)		
40 and above	14 (4.17)	11 (78.57)	3 (21.43)		
Marital status					
Single	51 (15.18)	37 (72.55)	14 (27.45)	0.28	0.868
Married	263 (78.27)	193 (73.38)	70 (26.62)		
Co-habiting	22 6.55)	15 (68.18)	7 (31.82)		
Religion					
Christianity	317 (94.35)	232 (73.19)	85 (26.81)	0.21	0.650
Islamic	19 (5.65)	13 (68.42)	6 (31.58)		
Highest level of education					
No formal education	6 (1.79)	4 (66.67)	2 (33.33)	3.78	0.287
Primary	81 (24.11)	61 (75.31)	20 (24.69)		
Secondary	93 (27.68)	61 (65.59)	32 (34.41)		
Tertiary	156 (46.43)	119 (76.28)	37 (23.72)		
Employment status					
Unemployed	26 (7.74)	17 (65.38)	9 (34.62)	2.49	0.647
Student	17 (5.06)	12 (70.59)	5 (29.41)		
Government worker	112 (33.33)	87 (77.68)	25 (22.32)		
Private firm worker	25 (7.44)	17 (68.00)	8 (32.00)		
Self-employed	156 (46.43)	112 (71.79)	44 (28.21)		
Gestational period					
First trimester	43 (12.8)	29 (67.44)	14 (32.56)	0.99	0.608
Second trimester	96 (28.57)	69 (71.88)	27 (28.13)		
Third trimester	197 (58.63)	147 (74.62)	50 (25.38)		

### 3.6 Association between practices and the occurrence of VVC

A significant association was found between antibiotic use and the occurrence of vulvovaginal candidiasis as shown in Table 5. No significant association was found between the other variables and the occurrence of the infection.

**Table 5:** Association between practices and occurrence.

Characteristics	Confirmed cases			*χ*2	*p*-value
	Total (%)	Negative	Positive		
Practices					
Use feminine wash					
No	288 (85.71)	210 (72.92)	78 (27.08)	0.00	1.000
Yes	48 (14.29)	35 (72.92)	13 (27.08)		
Use perineal pads					
No	223 (66.37)	165 (73.99)	58 (26.01)	0.39	0.534
Yes	113 (33.63)	80 (70.80)	33 (29.20)		
Change underwear daily					
No	34 (10.12)	23 (67.65)	11 (32.35)	0.53	0.466
Yes	302 (89.88)	222 (73.51)	80 (26.49)		
Underwear fabric type					
Linen	7 (2.08)	5 (71.43)	2 (28.57)	0.03	0.987
Cotton	321 (95.54)	234 (72.90)	87 (27.10)		
Nylon	8 (3.38)	6 (75.92)	2 (25.00)		
Drying of underwear					
Bathroom	35 (10.420	24 (68.57)	11 (31.43)	5.52	0.063
Bedroom	57 (16.96)	35 (61.40)	22 (38.60)		
Under the sun	224 (72.62)	186 (76.23)	58 (23.77)		
Iron panty before use					
No	208 (64.20)	157 (75.48)	51 (24.52)	2.05	0.152
Yes	116 (35.80)	79 (68.10)	37 (31.90)		
Usually take antibiotics					
No	202 (60.12)	158 (78.22)	44 (21.78)	7.21	**0**.**007**
Yes	134 (39.88)	87 (64.93)	47 (35.07)		

The bold denotes significant statistical value.

### 3.7 Risk factors associated with the occurrence of VVC

Table 6 provides a summary of a bivariate and multiple logistic regression of risk factors and Occurrence of VVC. The variables were adjusted for each other. The significant variables from the chi-square analysis were considered, however, potential confounding variables like age, gestational period and employment status were included. Frequent antibiotic use was found as a significant factor in the occurrence of VVC. Individuals who used antibiotics frequently were twice more likely at risk of being infected compared to individuals who do not frequently use antibiotics. [AOR:2.25 95CI:1.33–3.79; *p*-value = 0.003].

**Table 6:** Bivariate and multiple logistic regression of risk factors and occurrence of VVC.

Characteristics	COR (95% CI)	*p*-value	AOR (95% CI)	*p*-value
Age				
16–29	1	0.054	1	0.114
30–39	0.61 (0.31–1.01)	0.371	0.64 (0.37–1.11)	0.536
40 and above	0.55 (0.14–2.06)		0.64 (0.16–2.59)	
Gestational period				
First trimester	1	0.597	1	0.819
Second trimester	0.81 (0.37–1.76)	0.336	0.91 (0.41–2.04)	0.597
Third trimester	0.70 (0.34–1.43)		0.82 (0.39–1.72)	
Employment status				
Unemployed	1	0.722	1	0.818
Student	0.79 (0.21–2.94)	0.194	0.86 (0.22–3.34)	0.346
Government worker	0.54 (0.22–1.37)	0.843	0.60 (0.22–1.66)	0.930
Private firm	0.89 (0.28–2.80)	0.506	0.99 (0.29–3.34)	0.861
Self- employed	0.74 (0.31–1.79)		0.89 (0.35–2.25)	
Frequent antibiotics use				
No	1	0.008	1	0.003*
Yes	1.94 (1.19–3.16)		2.25 (1.33–3.79)	

* Significant variable (*p*-value < 0.05).

## 4 Discussion

The study examined the knowledge, practices and occurrence of candidiasis among expectant mothers. It focused on the association between related practices and the prevalence of VVC. Practices such as the use of feminine hygiene wash, perineal pads and the use of antibiotics among others were ascertained. A strong association was found between frequent antibiotic use and the occurrence of candidiasis. A 27.1% prevalence of VVC was observed with a link to antibiotic use. However, there was no relationship found between knowledge and the other practices in relation to the infection.

A slightly elevated 27.1% prevalence was recorded compared to the 25% reported at Yemen (19), However, this occurrence is lower than the 29.2% prevalence indicated in a systematic review and meta-analysis conducted in Africa (20) and the 30.7% recorded in the Ho Municipality among gestational mothers (7). The dichotomy in these observed prevalences could be associated with the difference in sample size, study design and geographical location. While this study engaged 336 participants, that which was carried out in Yemen and the Ho Municipality involved 250 and 176 study subjects respectively. Given that VVC is common infection among pregnant women, there is a need to heighten awareness and screening of such mothers in the current study jurisdiction. This will foster early case detection and treatment to aid the mitigation of associated discomfort and the risk of complication during pregnancy.

An association was found between antibiotic use and the occurrence of VVC. This conforms with some recent studies which found associations between antibiotic usage and the prevalence of the infection. A study by Dou, Li (21) indicated that the widespread use of antibiotics contributes to an increased prevalence of Candida vaginal infection. Their study identified pregnancy, diabetes mellitus, contraceptive and antibiotic use as predisposing factors for the infection. Also, reports from another study indicated that about 28%–33% of women on antibiotic therapy develop symptomatic genital candidiasis (14). Again, a retrospective study conducted between 2014 and 2016, revealed that the use of gynecological antibiotics, systemic antibiotics, oral contraceptives as well as vaginal contraceptives were associated with an increase in the risk of vulvovaginal candidiasis (2). Another study characterized antibacterial therapy, whether administered systemically or locally to the vagina, as the most common and predictable cause or triggering factor for symptomatic VVC (15). It is therefore evident that, antibiotic use has an impact on the occurrence of VVC in most cases Hence, patients must be educated on the potential side effects of antibiotics, including the risk of candidiasis, and encourage them to take antibiotics only as prescribed and to complete the full course.

The study did not find the use of perineal pads, feminine hygiene wash, regular changing of underwear, use of cotton underwear, drying underwear under the sun and ironing of underwear to be significantly associated with VVC among the study participants. This finding aligns with the results of a study on the impact of supportive nursing instructions on the recurrence of VVC during pregnancy. Their research similarly showed no notable association between practices such as using daily protective pads, wearing cotton underwear, changing wet underwear frequently, employing vaginal douching, performing vaginal douching during menses, and the recurrence of vulvovaginal candidiasis infection during pregnancy (22). This could be due to the fact that pregnancy significantly alters hormone levels, particularly estrogen and progesterone. These hormonal changes can make the vaginal environment more conducive to the overgrowth of Candida species, which is the primary cause of VVC. Such biological factors can outweigh the influence of personal hygiene practices, making them less significant in determining recurrence.

The question on the causative organism, thus fungi was missed by majority, 62.20% of the study participants referred to it as a bacterial infection though a good knowledge was recorded with other questions on knowledge. Burning sensation, itchy vagina and abnormal vaginal discharge was rightly associated by the study participants with the infection. Questions on signs and symptoms were answered correctly. On the contrary, a previous study recorded poor knowledge on the signs and symptoms among pregnant women (23).

Findings on knowledge, practices, and prevalence of candidiasis among pregnant women emphasize the necessity of addressing this widespread but often ignored condition in maternal healthcare. Our findings revealed that though there is good knowledge on the signs and symptoms, there still remains a gap on the knowledge of the causative agent.

### 4.1 Limitations of the study

Questions on practices were self-reported which may be prone to recall bias. Also, data collected did not cover the types of antibiotics frequently used and the duration of usage.

## 5 Conclusion

A high prevalence of VVC (27.1%) was reported among the expectant mothers visiting Ho Teaching Hospital for antenatal care. The findings thus suggests that candidiasis is a common condition among pregnant women, necessitating heightened vigilance in prenatal care and the creation of evidence-based therapeutic guidelines. Generally, these mothers had good knowledge on VVC, although most could not associate VVC as a fungal infection. The study identified antibiotics usage as a contributing factor to VVC, which may also lead to antimicrobial resistance. There is therefore the need for education on rational use of antibiotics among the study population. Since this study is limited in assessing the antibiotics categorisation and duration of usage, it is recommended that future prospective studies are conducted to address this limitation.

## Data Availability

The raw data supporting the conclusions of this article will be made available by the authors, without undue reservation.
